# Professional Cowardice

**Published:** 1879-12

**Authors:** 


### EDITORIAL.

### PROFESSIONAL COWARDICE.

If conscience doth make cowards of us all, surely ignorance has an equal power. Reputation may also be added to this category. If, perchance, by dint of long and earnest effort, a man has secured a high and enviable reputation in his profession, how he nurses it and coddles it! He is as tender of it as if it were a shorn lamb. The scriptural injunction, not to look behind us, is a good one. Rather let us look forward for new and greater successes, not permitting the recollection of past failures, or apprehensions of failure in the future, to diminish our zeal for a noble work, or lessen our usefulness for humanity's sake. No unmanly considerations should interfere with any and every reasonable effort to alleviate human suffering, and to save human life.

Professional life is a constant combat. In disease we have an enemy to contend against which, too often, baffles the most consummate skill. A certain amount of risk, not only to life and health, as in treating contagious and infectious diseases, must be incurred, but also to reputation if failure to cure should be the poor reward for self-sacrificing labor and effort. The question may arise, whether we have a right to decline to assume the responsibility of a case, or perform an operation in which life may be imperiled, simply on account of the risk incurred to reputation in case of a fatal issue. For instance, we have observed in cases of membranous and diphtheritic croup, a hesitancy to resort to tracheotomy, even when it became apparent that the patient was hopelessly lost without a resort was made to this surgical procedure; yet the administration of muriate of ammonia and muriated tincture of iron was continued, and the unfortunate patient allowed to slowly choke to death, fearing, in case of failure, that ignorant people might say that the child died because the doctor cut its throat. In such cases it is acknowledged, even if life is not saved, the little sufferer is spared the horrible death from slow strangulation, with all its excruciating agonies. Such conduct deserves no other epithet than cowardice. If incompetent to perform an operation, it is the duty of the medical attendant to decline further responsibility, provided the case is at the same time relinquished. But if possessed of the requisite skill we have no right to decline, on account of the risk to reputation, to afford the patient the benefit of this dernier resort.

Not a score of years ago, the same position was taken by the profession in regard to the use of the forceps in difficult and lingering labors. Many entertained a great respect for this useful instrument at that period, yet failed to have the moral courage to use it on account of the risk to reputation, rather than the damage to human life from its application. To-day, the accoucheur who would incur the risk of fistula and inflammation, and the extreme exhaustion from lingering labors, rather than resort to the forceps, would be justly regarded as wanting in those elements of professional strength which should divest him of his diploma.

Virtue, not unlike manly courage, is often its own and only reward, and the effort required to bravely meet the difficulties so often encountered in the daily demands of professional life is an unerring test of fitness for its responsible duties. Medical men cannot escape the responsibilities peculiar to their noble calling even if they would. The profession holds them to the strict line of duty, and the public gives a critical judgment on the moral strength or the niggardly cowardice which, in the one case, makes them perform heroic services in behalf of suffering humanity, or, in the other case, causes them to skulk behind the phantom of reputation.

